# Self-Organized Nanostructure Modified Microelectrode for Sensitive Electrochemical Glutamate Detection in Stem Cells-Derived Brain Organoids

**DOI:** 10.3390/bios8010014

**Published:** 2018-02-05

**Authors:** Babak Nasr, Rachael Chatterton, Jason Hsien Ming Yong, Pegah Jamshidi, Giovanna Marisa D’Abaco, Andrew Robin Bjorksten, Omid Kavehei, Gursharan Chana, Mirella Dottori, Efstratios Skafidas

**Affiliations:** 1 Centre for Neural Engineering, The University of Melbourne, Melbourne, VIC 3053, Australia; rachael.chatterton@florey.edu.au (R.C.); jasony1@student.unimelb.edu.au (J.H.M.Y.); golpeg@yahoo.com (P.J.); gchana@unimelb.edu.au (G.C.); sskaf@unimelb.edu.au (E.S.); 2 The Department of Electrical and Electronic Engineering, The University of Melbourne, Melbourne, VIC 3010, Australia; 3 ARC Centre of Excellence for Integrative Brain Function, The University of Melbourne, Melbourne, VIC 3010, Australia; 4 The Department of Biomedical Engineering, The University of Melbourne, Melbourne, VIC 3010, Australia; gdabaco@unimelb.edu.au; 5 The Department of Anaesthesia & Pain Management, Royal Melbourne Hospital, Parkville, VIC 3050, Australia; andrew.bjorksten@mh.org.au; 6 Faculty of Engineering and Information Technology, The University of Sydney, Sydney, NSW 2006, Australia; omid.kavehei@sydney.edu.au; 7 Department of Medicine, Royal Melbourne Hospital, The University of Melbourne, Melbourne, VIC 3050, Australia; 8 Illawarra Health and Medical Research Institute, Centre for Molecular and Medical Bioscience, University of Wollongong, Wollongong, NSW 2522, Australia

**Keywords:** microelectrodes, electrochemical biosensors, amperometry, glutamate, human embryonic stem cells, organoids

## Abstract

Neurons release neurotransmitters such as glutamate to communicate with each other and to coordinate brain functioning. As increased glutamate release is indicative of neuronal maturation and activity, a system that can measure glutamate levels over time within the same tissue and/or culture system is highly advantageous for neurodevelopmental investigation. To address such challenges, we develop for the first time a convenient method to realize functionalized borosilicate glass capillaries with nanostructured texture as an electrochemical biosensor to detect glutamate release from cerebral organoids generated from human embryonic stem cells (hESC) that mimic various brain regions. The biosensor shows a clear catalytic activity toward the oxidation of glutamate with a sensitivity of 93 ± 9.5 nA·µM^−1^·cm^−2^. It was found that the enzyme-modified microelectrodes can detect glutamate in a wide linear range from 5 µM to 0.5 mM with a limit of detection (LOD) down to 5.6 ± 0.2 µM. Measurements were performed within the organoids at different time points and consistent results were obtained. This data demonstrates the reliability of the biosensor as well as its usefulness in measuring glutamate levels across time within the same culture system.

## 1. Introduction

Glutamate is the principal excitatory neurotransmitter in the brain. In addition to its neuronal signaling role, glutamate also plays critical roles in cortical development, including migration and differentiation of cortical neurons. In contrast, high levels of glutamate may cause excitotoxicity and neuronal death that are associated with multiple neuronal diseases, such as ischemia [1,2], Parkinson’s disease [3,4], Alzheimer’s disease [5,6], and epilepsy [7,8,9].

These important roles of glutamate have generated a strong interest in the development of devices for the detection and monitoring of this amino acid. Moreover, given its fundamental role in corticogenesis and brain functioning, it would be valuable to measure glutamate levels at different stages of human brain development and within brain regions, with relevance to neurodevelopmental disorders. Neurospheres and cerebral organoids derived from human pluripotent stem cells (hPSC) consist of a heterogeneous mix of differentiating neural and glia cell types and can therefore mimic many of the dynamic processes occurring during brain development. This includes proliferation of neuroepithelial cells, neural/glial differentiation and cortical patterning. Using small molecule agonists and growth factors, neuronal cell fate within hPSC-derived neurospheres/organoids may also be biased to model different brain regions. hPSC-derived organoids can also be generated from patients suffering from neurodevelopmental or neurodegenerative conditions as potential disease models. Therefore, the development of a system that allows multiple and continuous measurements of glutamate levels within the same hPSC organoids cultured over time would greatly facilitate growing organoids with regional and disease specific characteristics.

Different methods have been established to determine glutamate levels, including optical methods [10,11], patch clamp [12], and microdialysis [13], as well as fluorometric [14], chromatographic [15], or spectrophotometric techniques [16,17]. Many of these techniques have intrinsic limitations, such as being time-consuming, requiring pre-treatment of the sample, being labour intensive, and requiring skilled handling. In contrast, electrochemical methods are considered as one of the most promising approaches because of low cost, simplicity, high sensitivity and specificity, which is ideal for point-of care, implantable and portable devices [18,19,20]. Amongst neurochemical devices, a range of amperometric biosensors have been developed based on loaded glutamate oxidase enzyme [21,22,23].

Here, we report the development and characterization of an enzyme based “micropipette-like” electrode with nanotextured probe, which is suitable for application in measuring glutamate levels within human embryonic stem cells (hESCs) derived organoids. We chose to test our microelectrode in a 3D organoid model in recognition of two-dimensional cell culture systems not being able to recapitulate the complexity of brain architecture and functioning. By creating nanostructures on the probe surface area we increased the binding capacity for the functional molecules to contribute in reactions at the surface, thus facilitating the acquisition and transportation of signals and improving sensitivity [24,25,26,27,28]. The performance of our nanotextured electrode was examined in hESC-derived organoids consisting of different neuronal populations that are representative of different brain regions and therefore likely differ in their glutamate levels. Measurements of glutamate levels were performed within the same organoids and across different timepoints in culture, thereby showing the multi-use application of the biosensor in live cell cultures. Overall, these studies demonstrate a novel approach for measuring dynamic changes of neurrotransmitter levels within hESC-derived cerebral organoids, a necessity for developing improved neurodevelopmental and neurological disorder models.

## 2. Materials and Methods

### 2.1. Materials

Dopamine (DA), ascorbic acid (AA), cysteine (Cus), uric acid (UA), serotonin, glucose, dihydroxyphenylacetic acid (DOPAC), alanine (Ala); arginine (Arg); asparagine (Asn); aspartic acid (Asp); glycine (Gly); histidine (His); trans-4-hydroxyl-l-proline (Hyp); isoleucine (Ile); leucine (Leu); lysine (Lys) and phosphate-buffered saline solution (PBS) all purchased from Sigma–Aldrich. Alexa Fluor^®^ 488 anti-rabbit-IgG antibody, DMEM/F12 medium, rabbit anti-v-Glut antibody (Abcam, Melbourne, Australia), mouse anti-GAD67 (EMD Millipore, Bayswater, Australia) and goat anti-rabbit (555 nm) purchased from Molecular probes, Life Technology.

### 2.2. Apparatus

Self-organized nanostructures were formed by means of reactive ion etching (Oxford Plasma Lab100, Oxford instruments, Abingdon, UK). Metallization was performed via electron beam evaporation (Intlvac Nanochrome, Fort Collins, CO, USA). The glass micro-needles were prepared through a flaming micropipette puller (P-100 Sutter Instrument, Novato, CA, USA). The amperometric experiments were computer controlled with data acquisition that was realized using a CHI760 interface, a low-noise damping potentiostat (600 E, CH Instruments, Austin, TX, USA). Surface analysis of fabricated microelectrodes was carried out using scanning helium ion microscopy (Nanofab Orion, Zeiss, Peabody, MA, USA). Fluorescence images were captured using a confocal microscope (Leica SP8, Wetzlar, Germany) with *Leica* Application Suite X software.

### 2.3. Biosensor Design and Fabrication

**Figure 1:**
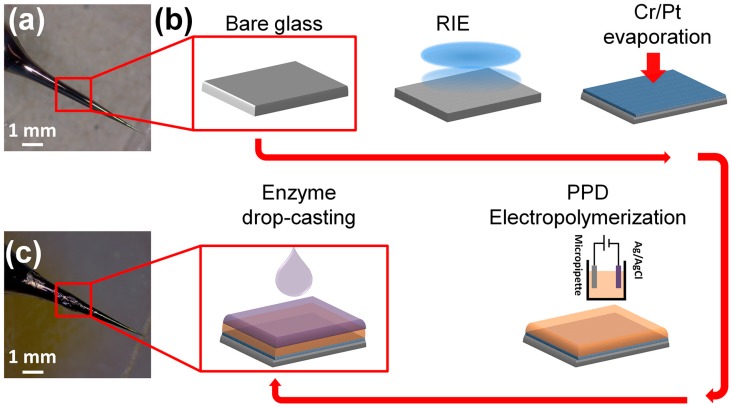
Biofabrication: (**a**) Top left: An optical image of a bare micropipette, used as the platform for microelectrode fabrication and glutamate biosensing. (**b**) Schematic illustration of microelectrode preparation. The microelectrode underwent RIE processing to increase the effective surface area by forming a nanostructured texture, followed by Cr and Pt deposition to form a signal transducer. The microelectrode was then modified by an electropolymerized PPD and covered by enzymatic film. (**c**) bottom left: An optical image of the final microelectrode covered by GluOx layer ready for amperometric measurements.

Figure 1 shows a schematic illustration of the biosensor fabrication process. The glass micro-needles were prepared by pulling borosilicate glass capillaries (i.d. 1.16 mm, 75 mm long, SDR scientific) through a flaming micropipette puller as shown in Figure 1a. The micropipettes with suitable geometry (smoothly tapered with tip size of 10 µm) were fabricated by adjusting the pulling program. The glass surfaces were cleaned with acetone, 2-propanol, deionized water and loaded into a reactive ion etcher (RIE) chamber to develop self-organized nanostructures under pseudo Bosch process [29]. The RIE was carried out for 20 min at RF/ICP powers of 600 W/30 W and etching gas (SF_6_: 100 sccm and O_2_: 10 sccm) while the chamber pressure stabilized at 20 torr.

**Figure 2:**
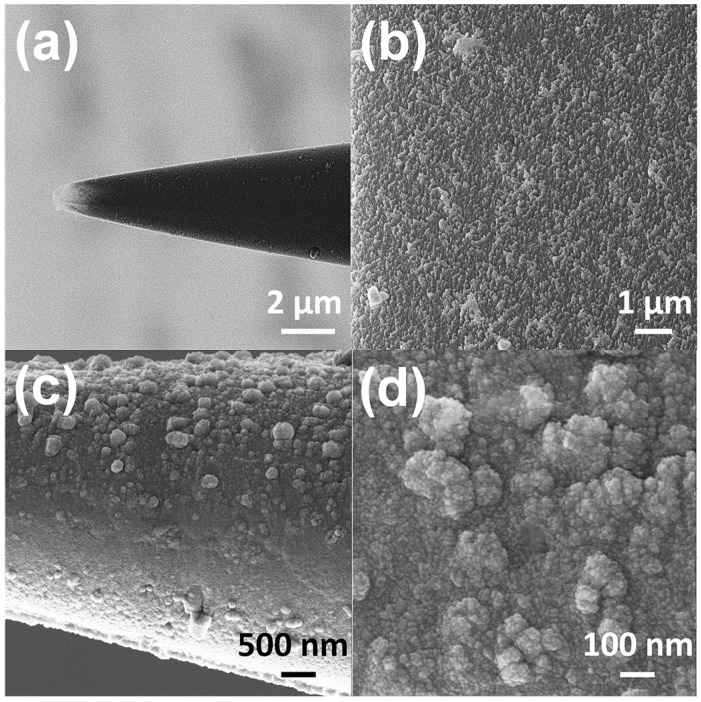
HIM imaging of a microelectrode (**a**) Overall view of the tip of a microelectrode used as probe for glutamate detection in hESC. (**b**) High resolution image of self-organized nanostructures on the surface of the probe. (**c**) The structural rearrangement of the probe after Cr/Pt deposition (**d**) High resolution image of the morphology of Pt coated nanostructures on the probe.

A helium ion microscope (HIM) equipped with charge cancellation system was used to observe and inspect the probe surface area of the microelectrode. The HIM was armed with a very low voltage electron gun (flood gun) to compensate for positive surface charge accumulation on the bare glass surface. During imaging the electron beam energy and the *X* and *Y* deflectors were adjusted correspondingly to ensure the best possible image. Figure 2a shows the HIM viewgraph of the probe and high-resolution image of nanostructured surface morphology (Figure 2b). A conductive thin film of Cr/Pt (30 nm/70 nm) was deposited on micropipettes by e-beam evaporation to act as the electrochemical transducer (Figure 2c,d). The micropipettes were vertically mounted on a rotation stage in deposition chamber and positioned above the vapor source to achieve uniform metallic coating at the tip of electrodes. The rotation speed of 20 rpm was maintained throughout the evaporation process.

Copper wires were then bonded to the end of platinum microelectrodes using conductive silver paste. Next, a layer of epoxy resin was coated onto the Pt micropipettes while leaving a millimeter of the tip exposed for enzyme coating.

### 2.4. Enzyme Immobilization on Microelectrodes

A surface treatment method was developed to immobilize glutamate oxidase onto the surface of the microelectrode tip/probe. First poly(m-phenylenediamine), PPD, was electropolymerized onto the tip of the microelectrode using an amperometric process. The electropolymerization is the most common technique for applying polymer films as it allows easy control of thickness of polymeric layer and a homogeneous coating onto electrodes with complex geometry [30]. A solution of PPD (100 mmol/L) was made up in 10 mL of a 0.01 M phosphate-buffered saline solution (PBS) with dissolution achieved by sonication at 25 °C for 25 min. The electro-oxidative polymerization was then performed by holding the potential at 0.7 V with reference to Ag/AgCl for 20 min followed by immobilization of glutamate oxidase (GluOx) via drop casting. The enzymatic solution consisted of 5 µL of phosphate buffered saline solution (PBS 0.01 M, pH 7.4) containing 10% BSA and 0.125% glutaraldehyde, which was mixed with 5 µL GluOx (50 U/mL). The exposed surface area was then drop coated repetitively (10 times with one-minute interval) with microdrops (~1 µL) of enzyme mixture using Eppendorf micropipette while the microelectrode turned over every time before drop casting to ensure a uniform coating. (Figure 1c). After the coating procedure, sensors were cured at least 24 h at 4 °C prior to use. A very thin yellow transparent layer could be seen under a microscope.

### 2.5. Cell Culture

This project was approved by the University of Melbourne Human Ethics Committee (#1545384). The hESC (WA-09, WiCell, Madison, SD, USA) cell line was cultured feeder free on vitronectin-coated plates using MTeSR-1 defined media according to the manufacturer’s instructions (Stem Cell Technologies, Tullamarine, Australia) and maintained at 37 °C and 5% CO_2_. Colonies were mechanically dissected every 7 days and transferred to freshly coated plates. Cell culture medium was changed every day. Neural induction and organoid formation of hESC were set up as described by Denham and Dottori, with some minor modifications [31]. HESC colonies were mechanically dissected into pieces approximately 0.5 mm in width and transferred to laminin-coated organ culture plates in ‘N2B27’ medium containing 1:1 mix of DMEM/F12 medium and neural basal media (Life Technologies/Invitrogen, Carlsbad, NM, USA) supplemented with 1% insulin/transferrin/selenium (Life Technologies/Invitrogen), 1% N_2_ supplement (Life Technologies/Invitrogen), 1% retinol-free B27 supplement (Life Technologies/Invitrogen), 0.3% glucose, 25 U/mL penicillin, and 25 μg/mL streptomycin (Life Technologies/Invitrogen). For differentiation to dorsal forebrain neurons, N2B27 defined medium was supplemented with the small molecules SB431542 (10 uM, Tocris, Bristol, UK) and LDN193189 (100 nM, KareBay Biochem, Monmouth Junction, NJ, USA) for 7 days. For differentiation to ventral forebrain neurons, N2B27 was supplemented with small molecules SB431542 (10 µM), LDN193189 (100 nM), and Smoothen Agonist (SAG; 400 nM, Merck Millipore, Bayswater, Australia) for 7 days. Following initial neural induction, colonies from dorsal or ventral forebrain neural inductions were treated with N2B27 medium supplemented with basic-fibroblast growth factor (bFGF; 20 ng/mL, Peprotech, Rehovot, Israel) for a further 7 days. Following 2 weeks of neural induction, neural progenitors were mechanically harvested and cultured in suspension in neural basal media supplemented with 1% insulin/transferrin/selenium, 1% N2 supplement, 1% retinol-free B27 supplement, 0.3% glucose, 25 U/mL penicillin, and 25 μg/mL streptomycin, bFGF, (20 ng/mL) and epidermal growth factor (EGF; 20 ng/mL, Peprotech, Rehovot, Israel) to promote organoid formation [32].

### 2.6. Immunoassay Analysis

Organoids were fixed with 4% paraformaldehyde and embedded in OCT for cryosectioning. Frozen cryosections were immunostained using rabbit anti-vGlut antibody or mouse anti-GAD67 followed by goat anti-rabbit (555 nm) or goat anti-mouse (488 nm) Alexafluor secondary antibodies, respectively. All samples were counterstained with 4’,6-diamidino-2-phenylindole (DAPI; 1 µg/mL, Sigma-Aldrich, Castle Hill, Australia).

### 2.7. HPLC Analysis

Glutamate analysis was performed using the isocratic HPLC method which utilized naphthalene-2,3-dicarboxylaldehyde (NDA, Sigma-Aldrich, Castle Hill, Australia) derivatisation and subsequent fluorescence detection [33,34,35]. ESC-derived organoids were lysed by sonication (50% Amplitude, 3 pulses at 5 s with 25 s intervals) in 2% EDTA, 0.1 M Acetic Acid buffer and then centrifuged at 10,000 rpm for 5 min at room temperature. Supernatant was collected for HPLC analyses. Fifty µL of either standard solution or sample was added to 450 µL of borate buffer (0.1 M, pH 9.5), 100 µL of KCN (10 mM) and 100 µL of naphthalene-2,3-dicarboxylaldehyde (NDA, 6 mM) in a reaction tube, and the derivatization reaction allowed to proceed for 20 min in the absence of light. The fluorescent glutamate derivative (25 µL) was injected into the HPLC system, which consisted of a Waters 510 pump, a Bio-Rad AS-100 refrigerated auto sampler and an LDC FluoroMonitor 4100 programmable fluorescence detector at 420 nm excitation and 480 nm emission. The column was a 15 cm × 3.9 mm Waters Novapak C_18_ column, and the mobile phase was 20% acetonitrile in 50 mM KH_2_PO_4_ (pH 5.6) running at 2 mL/min. The method has within-day and between-day coefficients of variation of 1.97% and 2.94%, respectively, at 1 µM.

## 3. Results and Discussion

### 3.1. Principle of the Amperometric Glutamate Biosensor 

Nanostructured surfaces have excellent prospects for interfacing biological recognition events with electronic signal transduction where active enzymes sites are coupled directly with the nanostructure-based microelectrode resulting in direct electron transfer between the enzyme and signal transducer and thus enhanced biosensing properties. The nanostructured microelectrode provides an electro-active surface for enzyme immobilization with enhanced confirmation, orientation and biological activity.

Electrochemical detection involving the use of an oxidoreductase, i.e., GluOx, typically occurs according to the mechanism depicted in Figure S1. GluOx is one of the key elements when fabricating glutamate sensors for improving selectivity, stability and sensitivity towards glutamate. Glutamate oxidase (~140 kDa) has high substrate specificity to glutamate (k_cat_ = 75 s^−1^ and K_m_ = 0.23 mM) and high stability (thermal stability ~80 °C) [36,37]. It converts the substrate, glutamate, in presence of oxygen and water into α-ketoglutarate, hydrogen peroxide and ammonia. The hydrogen peroxide produced can be easily detected amperometrically [23]. Following the enzymatic reaction as described below, conversion of 1 M of glutamate will ideally generate 1 M of H_2_O_2_.

$$L-\mathrm{glutamic}\;\mathrm{acid}\;+\;H_{2}O\;+\;O_{2}\overset{\mathrm{GluOx}}{\to }\alpha -\mathrm{ketoglutarate}\;+\;NH_{3}\;+\;H_{2}O_{2}$$

The hydrogen peroxide then reacts electrochemically on the Pt electrode as shown below:

H_2_O_2_ → 2H^+^ + O_2_ + 2e^−^

For an ideal immobilization, the GluOx needs to bind to the electrode firmly and maintain its structure and function properly after immobilization. The most common method for fabricating glutamate biosensors is crosslinking which uses reagent bovine serum albumin (BSA) as the enzyme stabilizer and glutaraldehyde as the crosslinker [21]. BSA is often included in the cross-linked matrix to protect enzyme activity by creating a 3D network for enzyme attachment [1,38]. On the other hand, glutaraldehyde is reactive towards the amine groups of lysine residues located mainly on protein surfaces and therefore can crosslink and immobilize GluOx and BSA on the microelectrode surface.

Since other biogenic substances can also be oxidized at the microelectrode probe surface, it was shielded with a permselective membrane to prevent these substances from approaching the signal transducer (platinum layer) [39]. Therefore, prior to surface functionalization, PPD was electropolymerized on the tip of the microelectrode using an amperometric process. The enzymatic layer was then formed by drop-casting a solution comprising BSA, glutaraldehyde and GluOx. The enzymatic layer, GluOx, was immobilized by drop-casting of 10 µL of an enzyme solution containing 10% BSA and 0.125% glutaraldehyde mixed with 0.5 µL GluOx (1 U/µL) on the tip of a borosilicate glass capillary at room temperature and then drying naturally for ~20 min.

### 3.2. Glutamate Biosensor Characterisation, Sensitivity and Selectivity Analysis

To address the analytical applicability of the Pt/PDD/GluOx coated microelectrodes we investigated the electrocatalytic activity of these microelectrodes by measuring the current response at different glutamate concentrations in PBS. Amperometry experiments were conducted using a standard three-electrode set-up with Ag/AgCl as the reference and a platinum wire as the counter electrode. These experiments were computer controlled with data acquisition that was realized using a CHI760 interface, a low-noise damping potentiostat. The biosensors were initially calibrated as shown in Figure S2a. As described in other publications, electrochemical oxidation of H_2_O_2_ is achieved above +0.2 V [40,41,42]. Therefore, the chronoamperometric responses of the microelectrode were recorded during the successive addition of 5.0 µM and 100 µM glutamate in 1 M PBS at an applied potential of +0.7 V vs. Ag/AgCl which is optimal for H_2_O_2_ detection [40]. It was observed that the microelectrode could catalytically detect generated H_2_O_2_ through electrochemical transducer. To quantify the amperometric response, a calibration curve for each biosensor was created linking the current response to the glutamate concentration (Figure S2b). It was found that the biosensors responded linearly (R^2^ = 0.968 ± 0.007) to glutamate over the concentration range between 5 µM and 0.5 mM, with a LOD (3× standard measurement error bar of the baseline). Glutamine, of 5.6 ± 0.2 µM. indicating suitability for detection of low glutamate levels in many biological applications. Using the linear response range, the sensitivity was determined to be 93 ± 9.5 nA·µM^−1^·cm^−2^ by the slope of the calibration curve for the glutamate biosensor. This is higher than a large number of reported glutamate oxidase based sensors where the sensitivity varies between 2.15 and 65 nA·µM^−1^·cm^−2^ [43,44]. This can be attributed to the high surface area of the nanostructure-based microelectrode.

Major challenges for enzymatic glutamate detection methods are electrochemically active interferents, which generate electrochemical signals at the same potential as glutamate. Therefore the biosensors were evaluated for interference at an applied potential of 0.7 V vs. the Ag/AgCl in 1 M PBS solution with continuous additions of 100 µM aliquots of dopamine (DA), ascorbic acid (AA), cysteine (Cus), uric acid (UA), serotonin, glucose, dihydroxyphenylacetic acid (DOPAC), alanine (Ala), arginine (Arg), asparagine (Asn), aspartic acid (Asp), glycine (Gly), histidine (His), trans-4-hydroxyl-l-proline (Hyp), isoleucine (Ile), leucine (Leu), lysine (Lys) and glutamate (Glut). It can be observed from the current response in Figure S2c, that only a significant signal was obtained for glutamate compared to the other amino acids. This implies that the Glu:H_2_O_2_ ratio i.e., the effectiveness of enzyme GluOx to convert glutamate to H_2_O_2_, was not affected by the presence of these potentially interfering substances.

### 3.3. Measurement of Glutamate within hESC-Derived Organoids 

**Figure 3:**
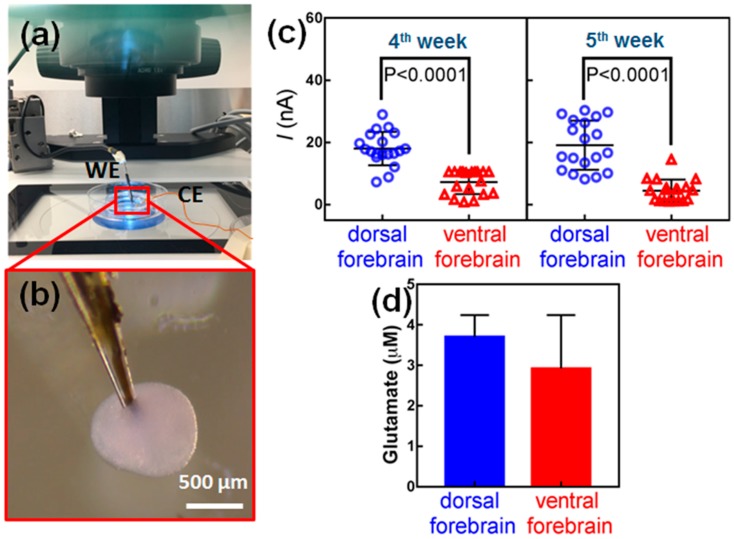
Glutamate measurements in hESC-derived organoids. (**a**) Image showing the set-up of equipment with dissecting microscope, organoid culture dish and microelectrode on holder attached to a micromanipulator. (**b**) The microelectrode is first located outside the organoid with an angle of about 40° against the culture dish. It is then gently forced into the organoid. (**c**) Biosensor measurements of glutamate levels in dorsal and ventral hESC-derived organoids cultured. Measurements were performed in the same organoids at 4 weeks and 5 weeks in culture. At least 20 organoids were examined for each experiment. *p* < 0.0001, Mann–Whitney U test. (**d**) HPLC analyses of glutamate.

We and others have established protocols for generating organoids from hESC that consist of cortical dorsal and ventral neural progenitors, which give rise to glutamatergic and GABAergic neurons, respectively [45,46,47]. Figure S3 shows glutamatergic and GABAergic neurons by means of an immunostaining method using rabbit anti-vGlut antibody or mouse anti-GAD67 followed by goat anti-rabbit (555 nm) or goat anti-mouse (488 nm) Alexafluor secondary antibodies. Since dorsal and ventral hESC-derived organoids mimic different brain regions with different cell populations, there may also be differences in glutamate levels. To examine this hypothesis, an experimental setup was designed for glutamate measurement inside organoids based on a microelectrode biosensor, Leica M80 stereomicroscope with ESD design and Leica MATS heating system. In this apparatus, shown in Figure 3a, the amperometric measurements were carried out in a sterile biohazard fume hood under an upright zoom lens optical microscope. The microelectrode was mounted at an angle of 30–40 degrees from the petri dish and directed into the field of view of the microscope. Then, it was lowered gently and moved toward an organoid using a micromanipulator (SÜSS MicroTec AG, Garching, Germany). The microelectrode was then slid sideways until it pierced an organoid from the side as seen in Figure 3b and Figure S5. A potential of +0.7 V vs. Ag/AgCl was applied to the electrode to record chronoamperograms. The microelectrode stayed still for about 100 s to allow recording signals in response to glutamate concentration in the organoid before it was retracted. Three independent biosensor readings were taken for each organoid in at least 20 dorsal forebrain organoids and 20 ventral forebrain organoids.

When the microelectrode was inserted in the dorsal forebrain organoid, a strong peak current, in range of ~20 nA to ~30 nA was recorded through data acquisition system, which was attributed to the electrochemical oxidation of glutamate secreted in the organoids and consequent oxidation of H_2_O_2_ (Figure S5). In contrast, a very weak peak current was obtained after insertion of the microelectrode in a ventral forebrain organoid. This clearly revealed that a larger electrochemical response was caused by dorsal forebrain organoids presumably due to higher concentrations of glutamate. The recorded data for every organoid is averaged and presented in Figure 3c. The biosensor readings indicate that dorsal forebrain organoids have significantly higher glutamate levels compared to ventral forebrain organoids at four weeks in culture (Figure 3c, *p* < 0.0001). The same organoids were measured again at five weeks in culture and consistent results were obtained as shown in Figure 3c. These analyses were further supported by HPLC analyses of lysed organoids, showing higher levels of glutamate in dorsal forebrain relative to ventral (Figure 3d) [48].

## 4. Conclusions

In conclusion, we have developed a novel electrochemical glutamate biosensor for highly sensitive and selective detection of glutamate in stem cells-derived brain organoids. The self-organized nanostructure-textured probe with high surface area provides a higher surface concentration of released electrons at the probe during the electrochemical detection of H_2_O_2_. Under optimal conditions, the detection of glutamate can be achieved with a LOD of 5.6 ± 0.2 µM and sensitivity of 93 ± 9.5 nA·µM^−1^·cm^−2^, which is comparable to reported methods. (Table S1). Overall, these data demonstrate the robustness of the biosensor in detecting glutamate levels within stem cell-derived organoids, and can also be utilized to measure dynamic changes in glutamate levels across time in culture. It is noteworthy that the proposed electrochemical biosensing strategy has the advantages of ease of use and low cost. This study provides compelling evidence for the utility of biosensors in developing modelling of human brain function using stem cells.

## Supplementary Materials

The following are available online at http://www.mdpi.com/2079-6374/8/1/14/s1: Figure S1. Bio-detection principle, Figure S2. Electrochemical characterization of a glutamate biosensor, Figure S3. Immunostaining images of hESC-derived cortical organoids, Figure S4. HIM images of hESC-derived cortical organoids, Figure S5. Measurement of glutamate within hESC-derived organoids, Table S1: A comparison of recording characteristics of few microelectrodes for the detection of glutamate.
