# Supplementary material for: Self-Organized Nanostructure Modified Microelectrode for Sensitive Electrochemical Glutamate Detection in Stem Cells-Derived Brain Organoids
Source: Biosensors (Basel). 2018 Feb 5;8(1):14. doi: 10.3390/bios8010014 (PMC5872062; doi:10.3390/bios8010014)
Supplement: Supplementary file 1 [file biosensors-08-00014-s001.pdf]

biosensors

Supporting Information: Self-Organized Nanostructure Modified Microelectrode for Sensitive Electrochemical Glutamate Detection in Stem Cells Derived Brain Organoids

Babak Nasr, Rachael Chatterton, Jason Hsien Ming Yong, Pegah Jamshidi, Giovanna Marisa D'Abaco, Andrew Robin Bjorksten, Omid Kavehei, Gursharan Chana, Mirella Dottori, Efstratios Skafidas

Contents:

Figure S1: bio-detection principle
Figure S2: electrochemical characterization of a glutamate biosensor
Figure S3: Immunostaining images of hESC-derived cortical organoids
Figure S4: HIM images of hESC-derived cortical organoids
Figure S5: Measurement of glutamate within hESC-derived organoids
Table S1: A comparison of recording characteristics of few microelectrodes for the detection of glutamate

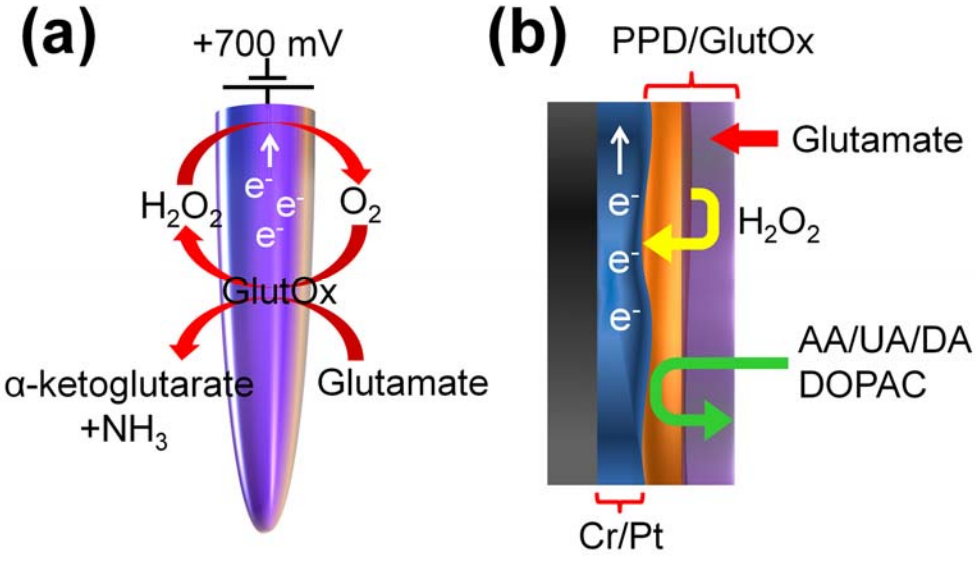

Figure S1. bio-detection principle. (**a**) schematic representation of enzymatic reaction that allows detection of glutamate at the microelectrode: glutamate is oxidized into α-ketoglutarate, ammonia and H_2O_2 by GluOx. H_2O_2 diffuses through the PPD layer and is oxidized by the Pt catalyst resulting in generation of electrons flowing into the microelectrode. (**b**) graphical view showing PPD layer acting as a diffusion barrier to biomolecule species. H_2O_2 can reach the electrode while larger molecules are rejected.

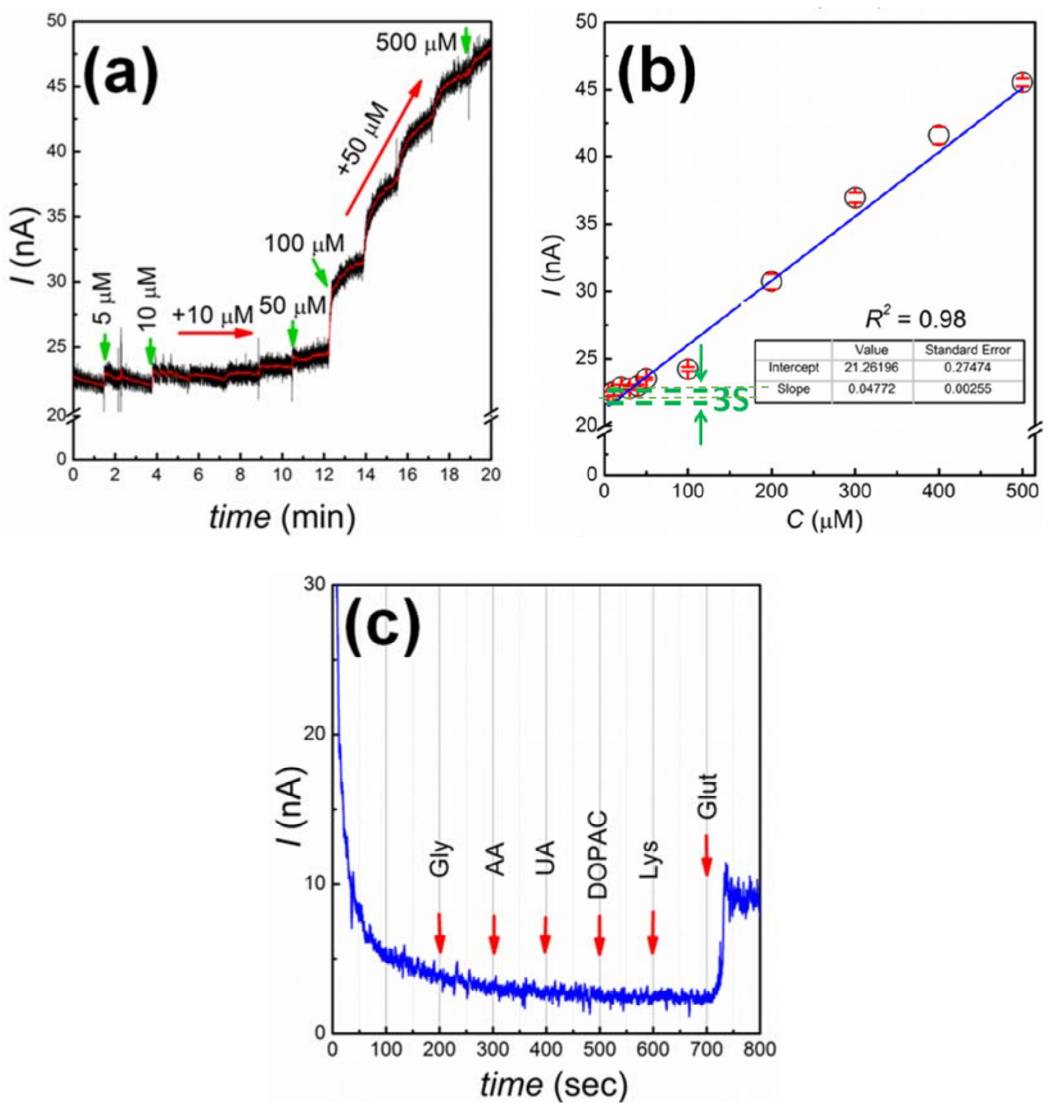

Figure S2. electrochemical characterization of a glutamate biosensor. (**a**), the amperometric response of the glutamate biosensor to successive addition of glutamate aliquots in the 1M PBS solution at the potential of 0.7 V vs. Ag/AgCl; (**b**), Corresponding calibration graph of the biosensor and regression analysis for sensitivity evaluation. The dashed lines indicate the limit of detection (LOD) obtained from three-times the standard measurement error bar of the baseline; (**c**), Current–time response curves in 1M PBS with successive additions of 100 μM glycine (Gly), Ascorbic acid (AA), uric acid (UA), dihydroxyphenylacetic acid (DOPAC), lysine (Lys) and glutamate (Glut) under 0.7 V electrode potential vs. Ag/AgCl.

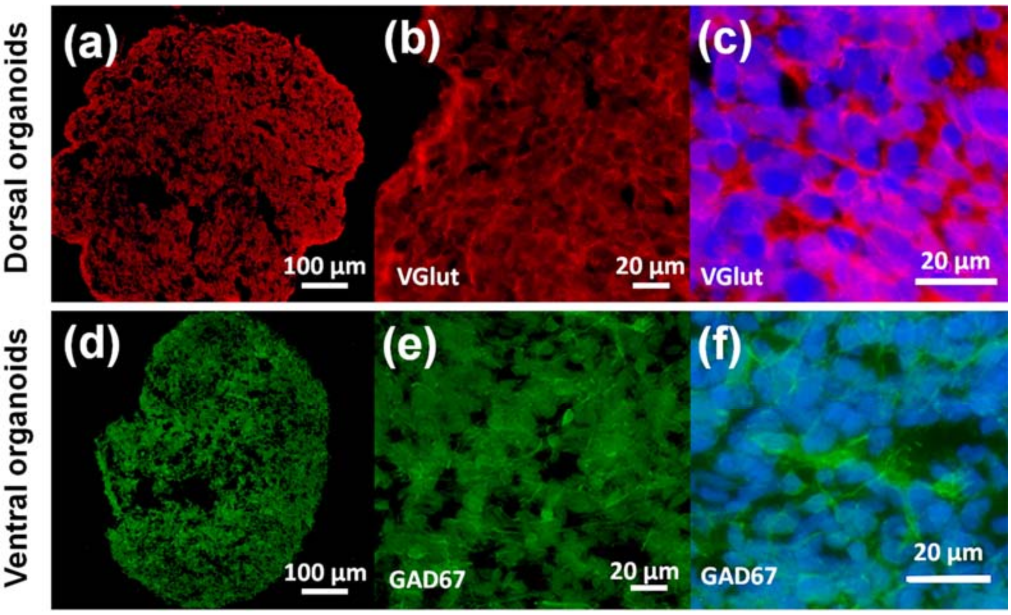

Figure S3. Immunostaining images of hESC-derived cortical organoids. (**a–c**), Dorsal forebrain organoids show strong expression of the glutamatergic marker, vGlut (red). (**d–f**), Ventral forebrain organoids show expression of GABAergic neuronal marker, GAD67 (green). DAPI nuclei are shown in blue (**c, f**).

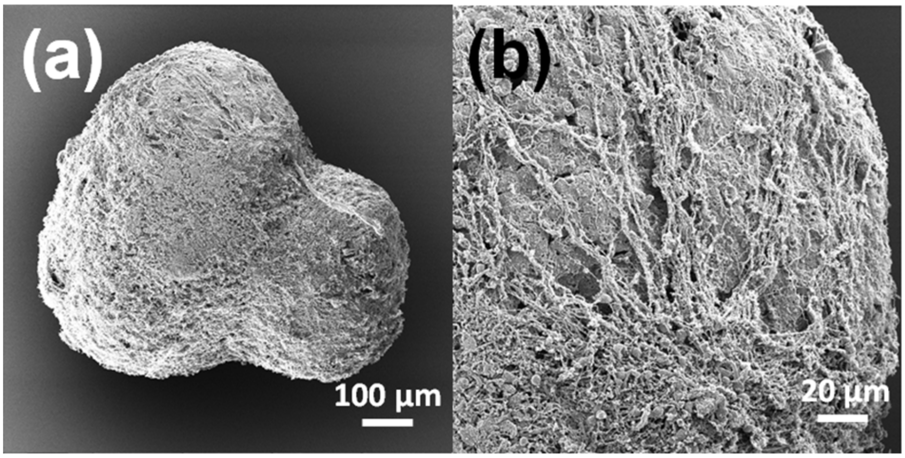

Figure S4. HIM images of hESC-derived cortical organoids. (a & b). a low and high magnification of a typical hESC-derived organoids. Organoids to be imaged were first washed with PBS and then fixed (2% glutaradelyde + 2% paraformaldehyde in 1M PBS, pH 7.4), for 20 min. Fixed samples were then washed twice with PBS and M-Q water. Next they were dehydrated through a graded ethanol series (30%, 50%, 75%, 85%, 95% and 100%: 15 minute washing). Fixed samples were then placed into an automated critical point drier (Balzers CPD 030, Hudson, USA) which was set to perform 10 exchange cycles of CO_2.After drying, the samples were carefully removed and adhered to double-sided carbon tabs on aluminum stubs and loaded into the microscope.

Neurospheres were imaged via the Helium Ion Microscope, HIM (Carl Zeiss, Orion Nanofab, Peabody MA, USA) operating at an accelerating voltage of 30 kV and a beam current of approximately 0.5 pA. No further metallic coating was performed since the HIM is armed with a very low voltage electron gun (flood gun) to compensate for positive surface charge accumulation on the insulating biological samples. Under these experimental conditions, no obvious beam damage or change in morphology was observed on the surface of neurospheres.

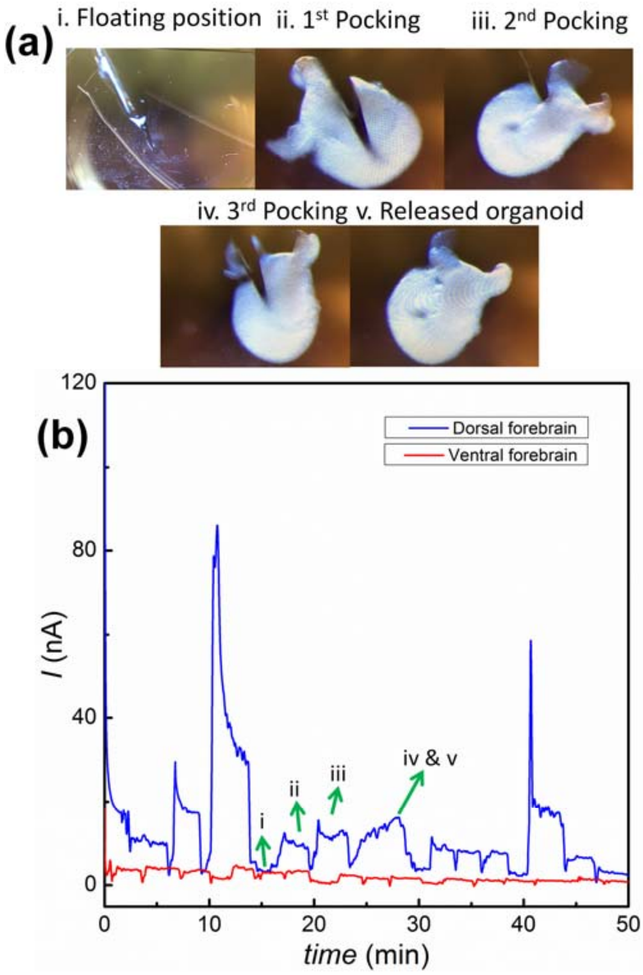

Figure S5. Measurement of glutamate within hESC-derived organoids. A. the optical images of the microelectrode at deferent positions in i) floating position, ii) first penetration iii) second penetration iv) third penetration and v) after completion of measurement in an organoid. The microelectrode stayed still for about 100 sec to allow recording signals in response to glutamate concentration. **B.** The chronoamperometric responses were taken for each organoid in at least 20 dorsal forebrain organoids and 20 ventral forebrain organoids. The biosensor readings for each organoid after each measurement were averaged and presented in Figure 3B.

Table S1. A comparison of recording characteristics of few microelectrodes for the detection of glutamate.

Electrodes	Sensitivity / nA $\mu M^{-1} cm^{-2}$	LOD / μM	Linearity/ nA $\mu M^{-1} cm^{-2}$	Ref
Ni/NAE	65	135	0.8	[47]
Pt	38.1	2.5	0.998	[48]
Pt-AuNP TiO_2	194.6	1	--	[49]
Pd	39	0.5	0.999	[50]
Pt	100	---	0.85	[41]
Pt	93 ± 9.5	5.6 ± 0.2	0.980	This paper
